# A new method of intra-small intestinal ultrasonography using double-balloon enteroscopy

**DOI:** 10.1055/a-2603-5355

**Published:** 2025-06-13

**Authors:** Yafei Zhang, Lamei Wang, Lan Liu, Fan Wang, Qiu Zhao

**Affiliations:** 1 89674 Department of Gastroenterology, Zhongnan Hospital of Wuhan University, Wuhan, China; 2 Hubei Key Laboratory of Intestinal and Colorectal Diseases, Hubei Provincial Clinical Research Center for Intestinal and Colorectal Diseases, Wuhan, China; 3 575473 Department of Ultrasound, Hubei Provincial Hospital of Traditional Chinese Medicine, Wuhan, China

Intra-small intestinal ultrasonography (ISUS) performed during enteroscopy is a promising technique for characterization of small intestinal diseases. However, the use of ISUS is still infrequent 1 . Miniprobe endoscopic ultrasound (EUS) is usually performed with a 2-channel therapeutic endoscope; one is the working channel for passing the miniprobe and the other is the water-filling channel 2 . However, all the commercially available enteroscopes are designed with a single working channel, at present without the water-filling channel. Therefore, full water injection into the lesion site has to be done in advance through the working channel for ISUS. But the small intestine usually moves very quickly and the water is lost rapidly, which makes performing ISUS during enteroscopy difficult.

In our unit, ISUS was carried out using the DP-20L miniprobe (260-cm working length, 2.5-mm outer diameter, 20 MHz; InnerMed, Shenzhen, China) with an EN-580T enteroscope (double balloon-assisted, 200-cm working length, 3.2-mm working channel; FUJIFILM, Tokyo, Japan). Conventionally, a disposable latex balloon is mounted to the distal end of the EN-580T endoscope before each procedure, and simultaneously an external air pump is attached to the balloon port of the endoscope. To solve the problem of continuous water injection during ISUS examination, the water pump is attached to the balloon port of the endoscope, and the endoscope balloon removed ( Fig. 1 ). In vitro, the water injection rate can be up to 1 ml/s through this channel ( Video 1 ). In vivo, since the opening of this channel is about 1.5 cm behind the tip of the endoscope, the water injected through the channel often pools to the rear area of the targeted lesion, which decreases the inspection efficiency. In view of this problem, an extension tube for better water filling was designed. The tube was a cutting approximately 1.5 cm in length from the flexible tube of a disposable venous infusion needle, with both a horizontal tip and an oblique tip (PVC; Hongda, China). Subsequently, the tube was mounted to the distal end of the endoscope by adhesive tape, and the oblique end of the tube fastened to the opening of the air flow channel. Finally, the other end of the tube with the horizontal tip was excised flush with the endoscope tip ( Fig. 2 ). This method transformed the original air flow channel to a water-filling channel, enabling continuous water filling in the ISUS examination during enteroscopy ( Video 1 ). It should be noted that after the modification, the EN-580T double balloon-assisted endoscope was converted to a single balloon-assisted enteroscope and the corresponding insertion procedure needs to be used.

**Fig. 1:**
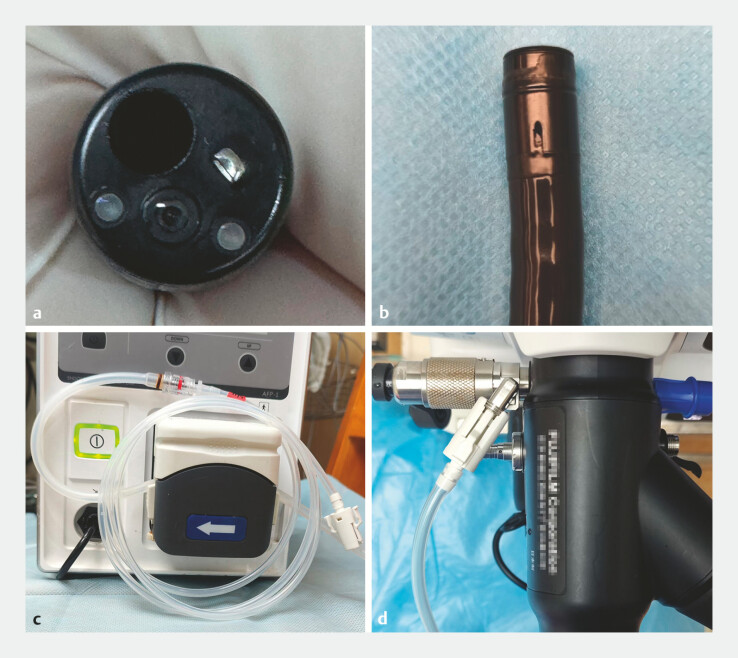
**a** No water-filling channel in the EN-580T endoscope. **b** The original air flow channel of the EN-580T endoscope. **c, d** The water pump was attached to the former balloon port of the EN-580T endoscope.

**Fig. 2:**
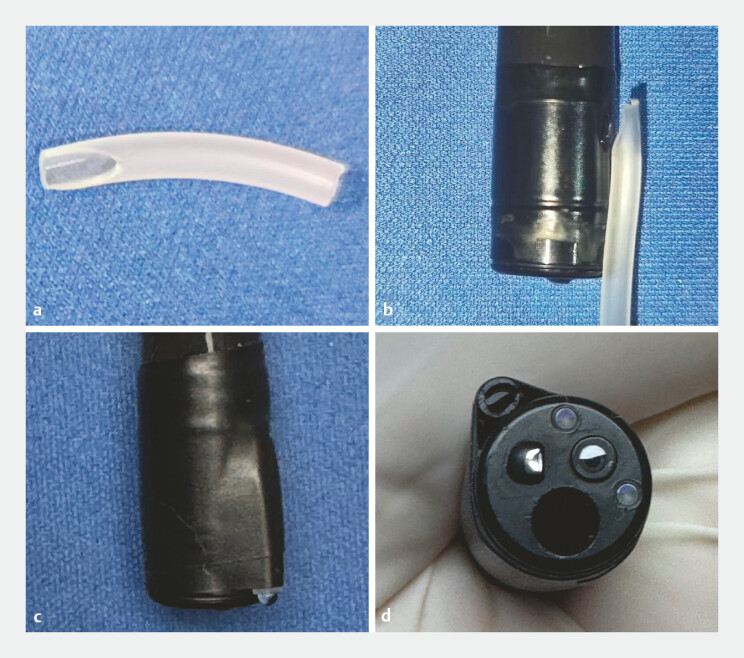
**a** The extension tube cutting from the flexible tube of a disposable venous infusion needle. **b** The oblique tip of the extension tube was fastened to the opening of the air flow channel. **c, d** Extension tube after tape fixation and excising.

This case reported a new method of intra-small intestinal ultrasonography with miniprobe. By transforming the air flow channel of the EN-580T endoscope to a water-filling channel, continuous water filling could be realized for intra-small intestinal ultrasonography using enteroscopy.Video 1

Endoscopy_UCTN_Code_TTT_1AP_2AD

## Notes

Endoscopy E-Videos https://eref.thieme.de/e-videos
*E-Videos* is an open access online section of the journal *Endoscopy* , reporting on interesting cases and new techniques in gastroenterological endoscopy. All papers include a high-quality video and are published with a Creative Commons CC-BY license. Endoscopy E-Videos qualify for HINARI discounts and waivers and eligibility is automatically checked during the submission process. We grant 100% waivers to articles whose corresponding authors are based in Group A countries and 50% waivers to those who are based in Group B countries as classified by Research4Life (see: https://www.research4life.org/access/eligibility/ ). This section has its own submission website at https://mc.manuscriptcentral.com/e-videos .
